# Parallel adaptations to nectarivory in parrots, key innovations and the diversification of the Loriinae

**DOI:** 10.1002/ece3.1131

**Published:** 2014-06-16

**Authors:** Manuel Schweizer, Marcel Güntert, Ole Seehausen, Christoph Leuenberger, Stefan T Hertwig

**Affiliations:** 1 Naturhistorisches Museum der Burgergemeinde BernBernastrasse 15, CH 3005, Bern, Switzerland; 2 Aquatic Ecology and Macroevolution, Institute of Ecology & Evolution, University of BernBaltzerstrasse 6, CH 3012, Bern, Switzerland; 3 Fish Ecology and Evolution, EAWAGSeestrasse 79, CH 6047, Kastanienbaum, Switzerland; 4 Department of Quantitative Economics, University of FribourgBoulevard de Pérolles 90, CH 1700, Fribourg, Switzerland

**Keywords:** Comparative methods, diet shift, digestive tract, evolutionary trait shifts, morphological adaptations

## Abstract

Specialization to nectarivory is associated with radiations within different bird groups, including parrots. One of them, the Australasian lories, were shown to be unexpectedly species rich. Their shift to nectarivory may have created an ecological opportunity promoting species proliferation. Several morphological specializations of the feeding tract to nectarivory have been described for parrots. However, they have never been assessed in a quantitative framework considering phylogenetic nonindependence. Using a phylogenetic comparative approach with broad taxon sampling and 15 continuous characters of the digestive tract, we demonstrate that nectarivorous parrots differ in several traits from the remaining parrots. These trait-changes indicate phenotype–environment correlations and parallel evolution, and may reflect adaptations to feed effectively on nectar. Moreover, the diet shift was associated with significant trait shifts at the base of the radiation of the lories, as shown by an alternative statistical approach. Their diet shift might be considered as an evolutionary key innovation which promoted significant non-adaptive lineage diversification through allopatric partitioning of the same new niche. The lack of increased rates of cladogenesis in other nectarivorous parrots indicates that evolutionary innovations need not be associated one-to-one with diversification events.

## Introduction

Although most flowering plants are pollinated by insects, a considerable number of tropical angiosperms are pollinated by birds and bats specialized on nectarivorous diets (Bawa 1990; Sekercioglu 2006; Fleming and Muchhala 2008). The associated ecological specializations resulted in several radiations of nectarivorous birds and bats in the tropics and subtropics (Fleming and Muchhala 2008). Nectarivory has evolved convergently in several groups of birds, with the Neotropical hummingbirds (Trochilidae, 325–340 species), the Australasian honeyeaters (Meliphagidae, 182 species) and the sunbirds (Nectariniidae, 132 species) of Africa and Australasia, representing three major radiations of nectarivorous birds. Additionally, nectarivory can also be found in several groups of parrots (Psittaciformes). Parrots represent one of the most species-rich clades of birds (Jetz et al. 2012). While they feed mainly on seeds and fruits, the chiefly Australasian Loriinae (lories) are specialized on a nectarivorous diet (Collar 1997; Rowley 1998). The lories consist of 53 species (Collar 1997) and are considered generalized flower visitors with eucalypts being a particularly important nectar source (Fleming and Muchhala 2008). Besides the lories, the swift parrot *Lathamus discolor* of Australia, the genus *Loriculus* of Australasia and Indo-Malaysia as well as the genus *Brotogeris* of the Neotropics are all supposed to depend on nectar as food (Homberger 1980; Güntert 1981; Forshaw 1989; Collar 1997). Their specialization to nectarivory has evolved in convergence to that of the lories (cf. Wright et al. 2008; Schweizer et al. 2010, 2011).

Nectar is a liquid food source rich in sugars, which account for almost 100% of its dry weight (e.g., Lüttge 1976; Gartrell 2000). However, it contains only a small amount of amino acids, too low to satisfy the nitrogen requirements of a bird (Martínez del Rio 1994). Therefore, nectarivorous birds have to rely on other nitrogen sources like insects or pollen (Richardson and Wooller 1990; Brice 1992; van Tets and Nicolson 2000; Nicolson and Fleming 2003). Several morphological and physiological specializations to nectarivory have been described in the various nectarivorous bird groups (Schlamowitz et al. 1976; Brown et al. 1978; Richardson and Wooller 1986; Casotti and Richardson 1993; Casotti et al. 1998; Schuchmann 1999; Gartrell 2000; Gartrell et al. 2000; Nicolson and Fleming 2003; Downs 2004) and the specialized bill structure of some hummingbirds is even considered a result of coevolution with the morphology of pollinated flowers (Feinsinger and Colwell 1978; Temeles and Kress 2003).

The adaptation to a new food source like nectar may be considered an evolutionary key innovation in the sense that it creates an ecological opportunity and promotes species proliferation associated with expansion into a previously unused niche (Vermeij 1995; Yoder et al. 2010). In nectarivorous parrots, this may be particularly true for the lories, which were found to be unexpectedly species-rich given their age compared to the remaining parrot lineages (Schweizer et al. 2011). Ecological opportunity may lead to strong directional selection and fast adaptation (Hunter 1998; Kassen 2009; Yoder et al. 2010). Indeed, several morphological specializations of the feeding tract have been described for nectarivorous parrots, which may have been essential for them to effectively feed on nectar and pollen. The lories in particular appear to have gastrointestinal tracts highly adapted to nectarivory (cf. Gartrell and Jones 2001). Both they and *Lathamus* have muscular tongues with a brush tip allowing them to rapidly harvest nectar (Churchill and Christensen 1970; Güntert and Ziswiler 1972; Richardson and Wooller 1990; Gartrell and Jones 2001). It was further reported that lories have shortened intestines, size-reduced gizzards with reduced muscularity and koilin layers as well as special adaptations in the esophagus, proventriculus and intestine (Güntert 1981; Richardson and Wooller 1990). The *Loriculus* species analyzed so far and *Lathamus* both shared some of the adaptations of the lories in the esophagus, proventriculus and intestine (Güntert 1981). However, they were found to have comparatively more muscular gizzards and longer intestines, probably allowing them to feed on hard food like insects or seeds (Güntert and Ziswiler 1972; Güntert 1981; Gartrell 2000; Gartrell et al. 2000). The morphological and ecological similarities among the different nectarivorous parrot groups may indicate parallel evolution driven by natural selection. However, data on the morphometrics of the digestive tract of parrots have never been analyzed with correction for phylogenetic non-independence, and the putative adaptations to nectarivory as described above have never been statistically assessed. Additionally, the comparisons of Richardson and Wooller (1990) and Gartrell et al. (2000) were based on limited taxon sampling.

In this study, we therefore tested whether the morphological variation found in continuous traits of the digestive tract of the nectarivorous parrots reflects phenotype–environment correlations as would be expected if some of this variation reflects morphological adaptations to a nectarivorous diet. We therefore applied a phylogenetic comparative approach using phylogenetic generalized least squares (PGLS) ANCOVA, with diet as a covariate in the model to test for phenotype–environment correlations. We moreover tested whether a subset of species in a phylogenetic tree shows a trait shift or evolutionary jump at the base of their clade. Since the lories apparently show the strongest dependence on nectar among all nectarivorous parrots, we tested if their diet specialization was associated with significantly hastened morphological evolution at the base of their radiation. All analyses were based on 15 continuous characters of the digestive tract and a broad taxon sampling of 78 parrot species consisting of representatives of all major groups. A phylogenetic hypothesis was obtained using three nuclear exons and one mitochondrial gene.

## Material and Methods

### Dissection and morphological measurements

Measurements of the digestive tract were taken from 354 individual parrots (Table S1). The data are from Güntert (1981), complemented with 15 additional species (19 individuals). Body mass was calculated for every species as the mean of the fresh dead or frozen and thawed specimens dissected. All weights were rounded to the nearest 0.1 g. For *Micropsitta finschi* and *Loriculus philippensis,* body mass values were taken directly from the literature (Mayr 1931; Rand and Rabor 1960) and an average value was calculated combining literature data and the fresh weight of other dissected specimens not used in this analysis. All measurements were either taken to the nearest mm using dividers for longitudinal measurements (length of esophagus, glandular stomach, and intestine) or to the nearest 0.1 mm using calipers under a dissecting microscope for the other traits. All the digestive organs were eventually fixed in buffered formalin (4%) and are stored in the vertebrate collection of the Natural History Museum Bern.

The digestive tract was removed from specimens and spread out by cutting through the mesenteria under a watery solution of 0.75% NaCl (isotonic for birds). When specimens had been preserved in formalin before dissection, it was no longer possible to straighten out the intestine, and the length had to be measured by means of a sewing thread, with which all the curvatures of the intestinal loops could be followed exactly. As parrots lack Brunner's glands (glandulae duodenales) and caeca (Ziswiler and Farner 1972; Güntert 1981), it is not possible to subdivide the intestine into different sections. Length of intestine was measured from the pyloric orifice of the gizzard to the rectal widening into the cloaca.

**Figure 1:**
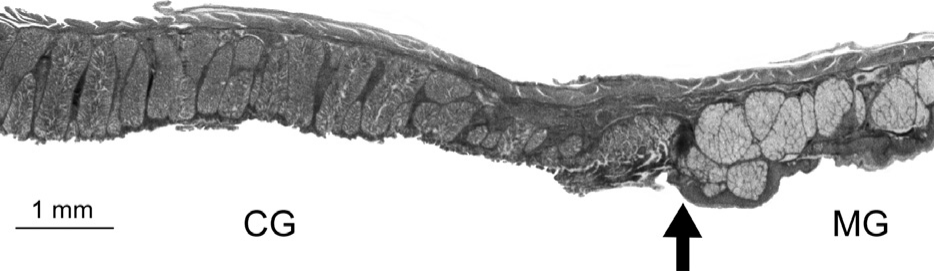
Longitudinal section through the epithelium at the border (arrow) between the esophagus (right) and the proventriculus (left) of *Psittrichas fulgidus*. The compound glands (CG) of the proventriculus can be clearly distinguished from the mucous glands (MG) of the lower part of the esophagus.

The esophagus is tripartite in parrots, consisting of a pars cervicalis (beginning at the posterior end of the larynx), the ingluvies (crop), and a pars thoracica that leads into the glandular stomach. Esophageal glands are restricted to the caudal area of pars thoracica. Length of the esophagus was defined as the distance from the caudal rim of the larynx to the border between esophageal and gastric glands (Fig. 1). To determine the extension of esophagus glands and the transition between the glandular part of the glandular stomach and its intermediate zone (see below), the digestive tube was cut open longitudinally.

**Figure 2:**
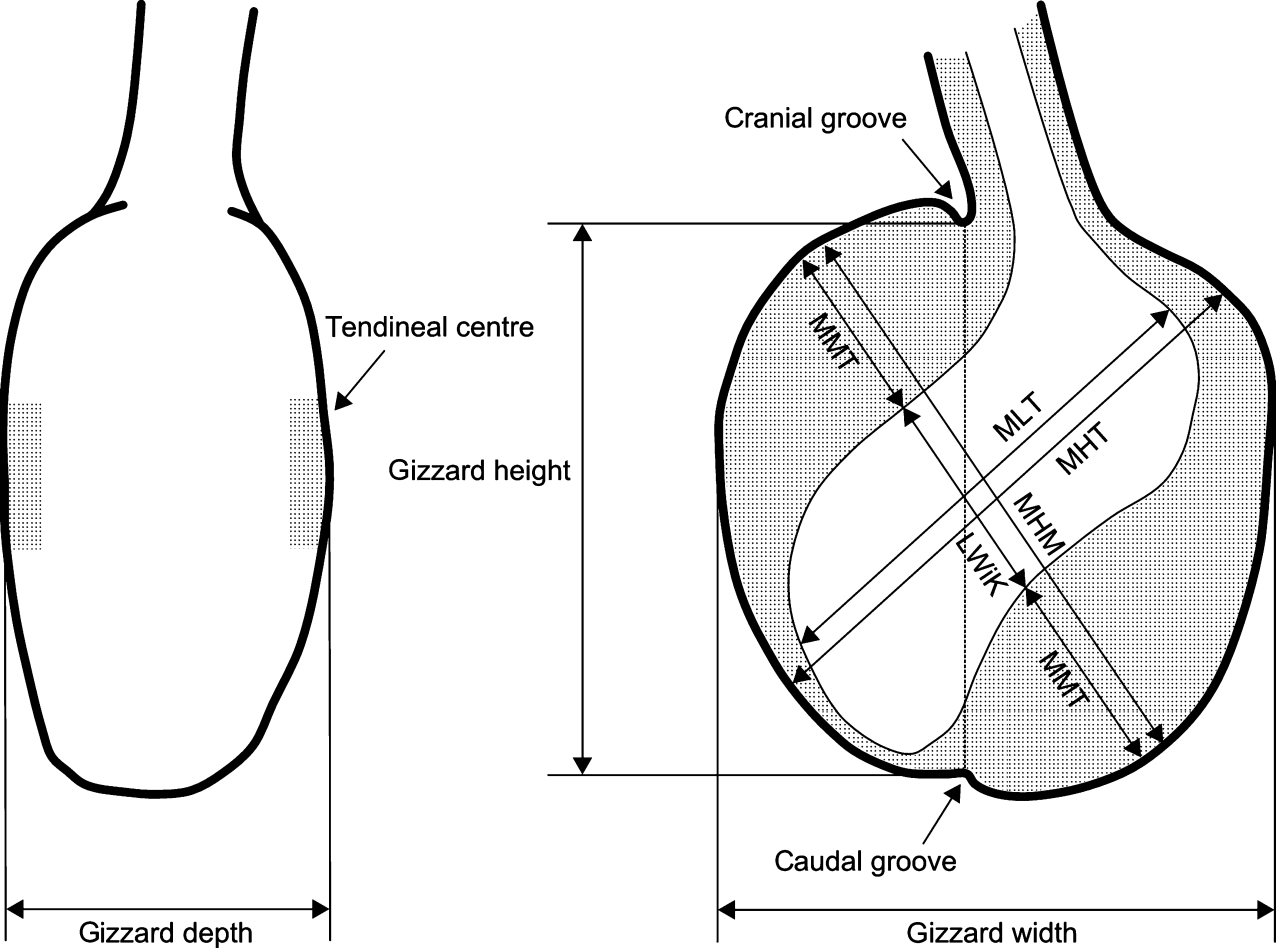
Schematic illustration of a caudal view and a transverse section along the median plane between the tendineal centers (right) of the gizzard. The measurements taken in this study are indicated. Modified from Ziswiler (1967).

The proventriculus or glandular stomach contains the gastric compound glands. This glandular part is followed by an intermediate zone (zona intermedia), lined with mucous glands. Total length of the proventriculus was measured from the first compound glands visible through the wall of the organ to the entrance into the gizzard. The caudal measuring point was the cranial groove, situated on the pyloric side of the proventricular tube (Fig. 2). The extent of the intermediate zone was computed as total length minus the glandular part (distance between the first and the most caudal compound glands).

**Figure 3:**
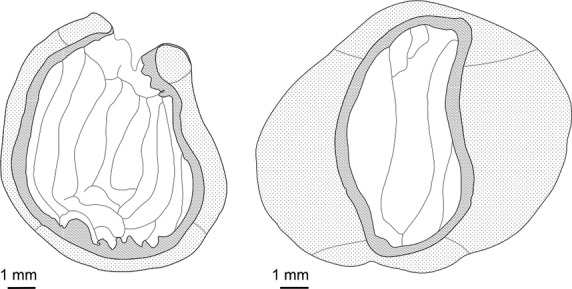
Schematic illustration of a transverse section through the gizzards along the median plane between the tendineal centres of a nectarivorous (*Vini australis*, left) and a granivorous parrot (*Neophema chrysostoma*, right).

The gizzard or muscular stomach has two opposing pairs of antagonistic muscles (Figs. 2, 3). Its inner surface is lined with a tough koilin membrane (Akester 1986), the cuticle of McLelland (1979), formed by mucosal glands. As external dimensions, we measured gizzard height (distance between the cranial and caudal groove), gizzard depth (minimum distance between tendineal centers on the two flat sides of the gizzard), and gizzard width (maximum distance at right angles of gizzard height) (Fig. 2). Maximum height at main muscles (MHM, thick muscle pair) and maximum height at thin muscles (MHT) were measured along the maximal extension of each muscle pair. Width of caudoventral thin muscle (WTM) was measured from the caudal groove to its outermost muscle bundles on the opposite side. Width of the lumen plus koilin layer (LWiK, including the distinctly visible tunica mucosa) was measured along the axis of the maximum height at main muscle MHM. Thickness of the two thick muscles (MMT) was calculated as the difference between MHM and LWiK. Lumen height (MLT) was quantified as the maximum distance between the opposite walls of the cranial and caudal sac.

### Phylogenetic analyses

To control for phylogenetic nonindependence in trait values, we reconstructed a phylogenetic hypothesis based on partial sequences of the three nuclear genes c-mos, RAG-1, and Zenk (second exon) and of the mitochondrial gene NADH dehydrogenase 2 (ND2) (Table S2). *Pitta* and *Falco* were used as outgroups and the tree was rooted with the latter taxon, but both were subsequently pruned from the tree before statistical analyses. Sequences of the three nuclear genes were taken from Schweizer et al. (2010) or newly generated following the laboratory protocol described in that study. ND2 sequences were taken from GenBank or generated using the primers MetL and ASNH for PCR amplification and sequencing from both sides (Tavares et al. 2006). The laboratory methods followed Schweizer et al. (2010) using the PCR Protocol of Tavares et al. (2006) for ND 2 with the annealing temperature set to 53°C. The alignment of the sequences was done manually with BioEdit 7.0.5.2 (Hall 1999). We checked individual sequences and the whole alignment further for quality by searching for apparent stop codons after the translation of sequences into amino acids. The final alignment was 4254 bp in length with 603 bp from c-mos, 1461 bp from RAG-1, 1149 bp from Zenk, and 1041 bp from ND2. It contained one indel of four amino acids for c-mos, one indel of three amino acids, and one indel of one amino acid for RAG-1, while for Zenk there were four indels of one amino acid and one indel of two amino acids. There were no ambiguously aligned amino acids.

Phylogenetic analyses were conducted with maximum likelihood (ML) using RAxML 7.0.4 (Stamatakis 2006). The program was run on the Web-server with 100 rapid bootstrap inferences with all free model parameters estimated by the software (Stamatakis et al. 2008). We tested different biologically relevant parameter settings for our concatenated data set corresponding to separate models of nucleotide substitution for genes and/or codon positions as estimated by RAxML. Akaike's information criterion (AIC) was used as a heuristic indicator for the fit of the different parameter settings (Akaike 1974), and using separate models of nucleotide substitution for the three codon positions of each gene separately was found to be the best-fitting model. This best-scoring ML tree with branch lengths of the best parameter setting was then used for further analyses.

### Phylogenetic generalized least squares (PGLS) ANCOVA

PGLS ANCOVA (Grafen 1989; Garland and Ives 2000) was used to estimate the relationships between our dependent and independent variables. This method incorporates a matrix of variance and covariance into the calculation of regression parameters based on the pattern of relatedness among species to account for the strong correlation in the error term (see also below). The variance-covariance matrix was calculated using the best-scoring ML tree. To assess the strength of phylogenetic signal in our data, we adjusted our model to include the parameter *λ*, which varies between 0 and 1 (Pagel 1997, 1999; Freckleton et al. 2002). Values of *λ* close to 1 imply that traits covary as assumed by a Brownian motion model with the original tree recovered, while values of *λ* close to 0 imply that there is almost no phylogenetic signal in the trait data, with the phylogenetic tree for the trait having a single polytomy at the basal node (Freckleton et al. 2002; Blomberg et al. 2003; Freckleton 2009). *λ* can be interpreted as having one component of the residuals evolving under a Brownian motion model, while another additive component has no phylogenetic correlation (Housworth et al. 2004; Lavin et al. 2008). Freckleton et al. (2002) showed that *λ* is statistically powerful in detecting whether the data show a phylogenetic signal, robust to incomplete phylogenetic information and that it performs better than Grafen's (Grafen 1989, 1992) *ρ* transformation. We implemented body mass as the independent variable, because gut measurements of birds are known to be allometrically related to body mass (e.g., Ricklefs 1996; Lavin et al. 2008), and the 15 morphometric distances described above were used as dependent variables. For all statistical analyses, both the independent and dependent variables were natural-log transformed.

To test for significant phenotype–environment correlations between the different traits in the digestive tract and nectarivory, we used diet (nectarivory of the lories, *Brotogeris*, *Lathamus,* and *Loriculus* versus the more general diets of other parrots) as a covariate in the model. We considered increasingly complex models, beginning with simple allometry between the dependent and independent variables, then diet was included as covariate using ANCOVA with different intercepts but the same slope and finally using ANCOVA with different intercepts and different slopes (i.e., diet body mass interactions). PGLS ANCOVA including the parameter *λ* were fitted by maximizing the restricted log-likelihood. To check for heteroskedasticity, plots of the residuals versus the fitted values were investigated. Akaike's information criterion (AIC) was used as a heuristic indicator for the fit of the different models (Akaike 1974) and we considered an increase in model-fit as significant when the reduction in AIC score in a more complex model was ≥4 (Burnham and Anderson 2002). We also compared the AIC of the models accounting for phylogenetic nonindependence with normal general least square approaches, which imply full independence of the data. All statistical analyses were performed in R 2.9.0 (R Development Core Team 2009) using the ape and nlme packages (Paradis et al. 2004; Pinheiro et al. 2009).

### Evolutionary trait shifts

We used an additional statistical approach to test for a significant shift in trait values at the base of a particular clade that cannot be explained by Brownian motion (Appendix S1). The underlying model for character evolution is a Brownian motion with sudden jumps which represent rapid changes. This so-called Lévy model has been used by many authors including Huelsenbeck et al. (2000), Uyeda et al. (2011) and Landis et al. (2012). If one assumes the null-hypothesis of no jumps this leads to a test statistic whose distribution is known and which thus allowed us to determine *P*-values in the context of a significance test. The basic model is a generalized linear model (GLM), similar to that described in Martins and Hansen (1997) and in Garland and Ives (2000):


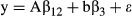
(1)

The vector y in equation (1) (see also Appendix S1) represents, for each of the *N* species, the logarithmic values of the measured traits which are considered as dependent variables. The matrix A consists of a column of ones (yielding the intercept of the regression line) and a column of logarithmic weights. The linear dependence between the logarithms of weight and measured trait for each species models the assumption of an allometric relationship between these quantities. The normally distributed error term *ε* reflects the hypothesis that under absence of selection the measured trait evolves according to a geometric Brownian motion (and thus the logarithm of the trait value follows a standard Brownian motion). The estimated phylogenetic relationship between the species again induces a strong correlation in the error term. In fact, the *(i*, *j)-*th element in the variance-covariance matrix *Σ*, corresponding to two species *i* and *j*, is proportional to the total length of the shared branches in the phylogenetic tree from root to the last common ancestor of *i* and *j*. The variance-covariance matrix was again calculated using the best-scoring ML tree.

The unknown factor of proportionality *σ* represents the drift speed of the Brownian movement and has to be estimated from the data. A *σ*-value of, for example, two would indicate that the relative rate of trait-change under neutral evolution is twice as fast as the rate of change in the genes used to estimate the variance-covariance matrix based on a phylogenetic tree.

Selection pressure on a subset of *K* species will result in a disproportionate change in the intercept value *β* for these species, a change that cannot be explained by Brownian motion (neutral evolution) alone. The additive term in equation (1) containing the parameter *β*_*3*_ models this possibility of selection pressure for the given subset of species. The null-hypothesis of no selection can be written as *β*_*3*_ = 0 and the alternative hypothesis corresponds to *β*_*3*_ ≠ 0. Large values of │*β*_*3*_│ will support the alternative hypothesis and result in large absolute values of the test statistic 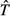 defined in (3). Under the null-hypothesis (absence of selection), the distribution of the test statistic is a *t*-distribution, see Theorem 1. Its proof is based on the technique of restricted least squares; the only nonstandard feature in our situation is the presence of correlation, called heteroskedasticity, in the error term. In order to reduce our model to the standard situation in restricted least squares, we have first to de-couple the error terms. The known distribution of the test statistic allowed us to report two-sided *P*-values. Low *P*-values support a rejection of the null hypothesis.

We used this approach to test if the lories show a trait shift or an evolutionary jump at the beginning of their radiation in the 15 measured continuous characters of the digestive tract (see above).

## Results

### Phylogenetic relationships

**Figure 4:**
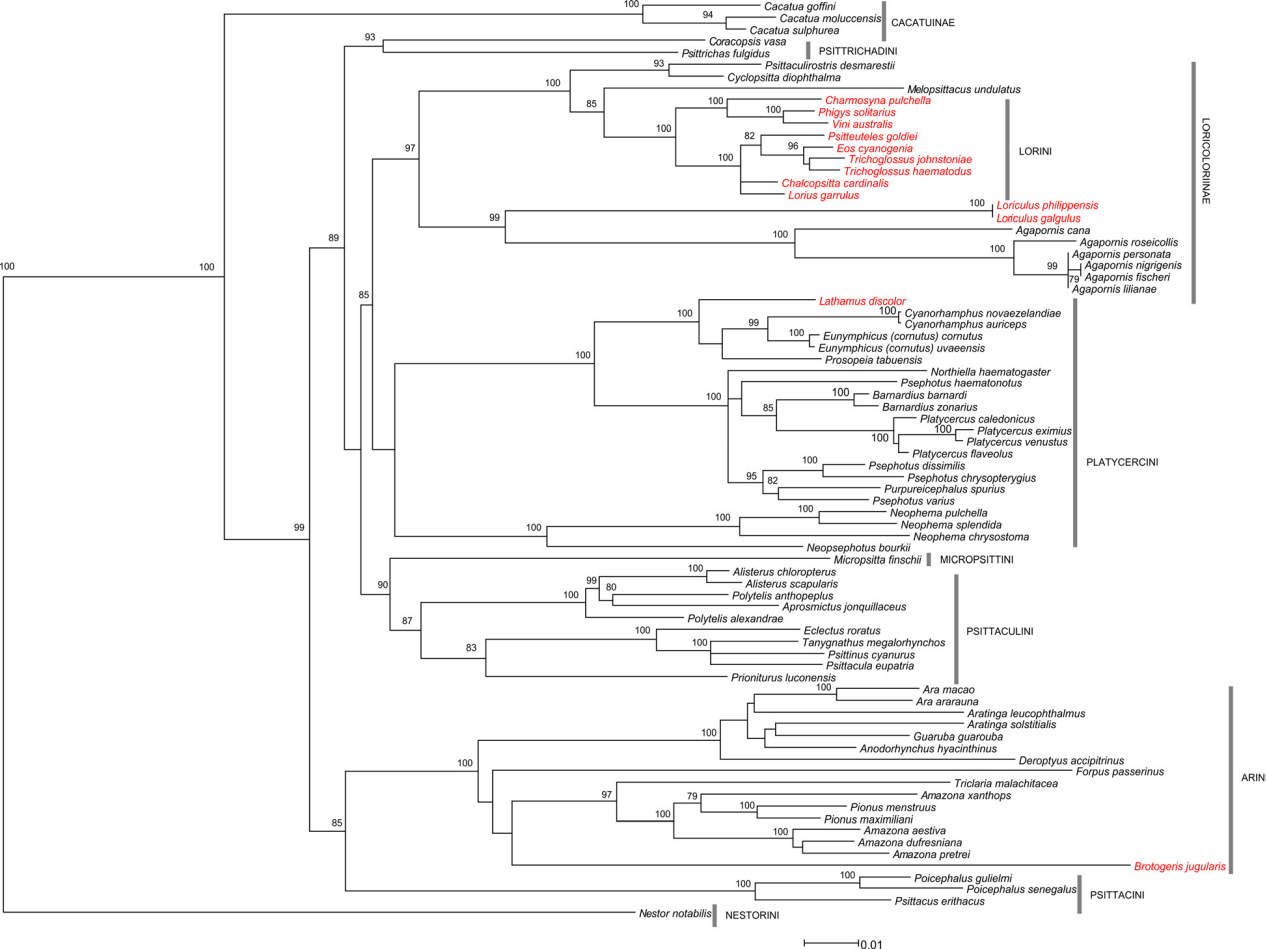
Best-scoring maximum likelihood tree including bootstrap values above 70% indicated at nodes. This tree was used for the formulation of the hypothesis of the variance-covariance matrix of the error terms in the regression analyses. Nectarivorous species are shown in red.

Our phylogenetic tree from the maximum likelihood analyses was in good agreement with Schweizer et al. (2010) (Fig. 4).

The lories were revealed to be a robustly supported monophyletic clade clustering as the sister group of *Melopsittacus undulatus*. The relationships within them were also highly supported with the exception of the monophyly of the genus *Trichoglossus* and the position of *Lorius* and *Chalcopsitta*. The division of the lories into two clades is in agreement with Wright et al. (2008). The position of *Loriculus* as the sister group of *Agapornis* was also robustly resolved and in agreement with other studies (Wright et al. 2008; Schweizer et al. 2010, 2011). Within Platycercini, the sister group relationship of *Lathamus* to a clade consisting of *Prosopeia, Cyanoramphus* and *Eunymphicus* was highly supported in congruence with other studies (Schweizer et al. 2010, 2011, 2013; Joseph et al. 2011). The sister group relationship of *Brotogeris* within the Arini was in congruence with Tavares et al. (2006) and Wright et al. (2008), but not supported in our data.

### PGLS ANCOVA

For linear body dimensions, a scaling with the 0.33 power of the body mass can be expected (Schmidt-Nielsen 1984). Indeed, we found scaling factors for all linear dimensions to lie around this expected value, ranging between 0.18 and 0.46. With the exception of gizzard lumen width including the koilin layer (LWiK), all other measurements showed a significant phylogenetic signal as indicated by lower AIC when phylogenetic information was incorporated in the model as compared to a generalized least squares regression (Table 1).

**Table 1:** Akaike's information criterion (AIC) for standard regressions (GLS) and phylogenetic generalized least squares (PGLS) including the fitted *λ*-values for the different models considered for each trait of the digestive tract. Increasingly complex models were tested, beginning with simple allometry between the dependent and independent variables, then diet (nectarivory) was included as covariate using ANCOVA with different intercepts but the same slope (Mass + Food) and finally using ANCOVA with different intercepts and different slopes (Mass × Food)

Trait	Model	AIC (GLS)	AIC (PGLS)	*λ*
Length of intestine	Simple allometry	38.906	−10.934	0.940
	Mass + Food	40.249	−6.783	0.942
	Mass × Food	43.976	−5.138	0.952
Length of esophagus	Simple allometry	−116.920	−125.082	0.511
	Mass + Food	−113.514	−119.100	0.498
	Mass × Food	−109.109	−112.824	0.497
Extension of esophagus glands	Simple allometry	91.273	45.734	0.977
	Mass + Food	48.695	40.746	0.952
	Mass × Food	46.092	42.497	0.615
Length of intermediate zone	Simple allometry	60.20076	20.7205	0.816
	Mass + Food	21.142	11.659	0.803
	Mass × Food	25.609	15.720	0.810
Length of proventriculus	Simple allometry	−72.200	−81.457	0.558
	Mass + Food	−81.509	−84.026	0.558
	Mass × Food	−76.821	−80.204	0.526
Gizzard height	Simple allometry	−45.0961	−78.692	0.766
	Mass + Food	−80.978	−84.985	0.602
	Mass × Food	−75.401	−79.171	0.602
Gizzard width	Simple allometry	−15.961	−54.317	0.774
	Mass + Food	−52.258	−58.246	0.652
	Mass × Food	−47.040	−52.899	0.661
Gizzard depth	Simple allometry	40.475	−21.107	0.914
	Mass + Food	−27.413	−34.577	0.804
	Mass × Food	−25.417	−31.727	0.769
Maximum gizzard height at main muscles (MHM)	Simple allometry	10.205	−28.377	0.763
	Mass + Food	−33.212	−36.807	0.589
	Mass × Food	−27.998	−31.641	0.593
Gizzard thickness at main muscles (MMT)	Simple allometry	169.006	102.775	0.938
	Mass + Food	103.458	89.283	0.898
	Mass × Food	106.376	91.087	0.917
Gizzard lumen width including koilin layer (LWiK)	Simple allometry	0.110	0.104	0.334
	Mass + Food	5.594	4.28	0.395
	Mass × Food	10.084	8.877	0.398
Gizzard width at caudoventral thin muscle (WTM)	Simple allometry	−37.786	−47.244	0.680
	Mass + Food	−33.593	−41.924	0.635
	Mass × Food	−32.005	−37.503	0.667
Maximum gizzard height at thin muscle (MHT)	Simple allometry	−37.340	−89.566	0.846
	Mass + Food	−82.851	−93.727	0.759
	Mass × Food	−76.966	−88.706	0.747
Maximum gizzard lumen at thin muscle (MLT)	Simple allometry	−30.160	−69.159	0.791
	Mass + Food	−62.319	−70.648	0.699
	Mass × Food	−56.805	−65.538	0.697
Gizzard mass	Simple allometry	130.144	75.418	0.880
	Mass + Food	75.552	66.876	0.787
	Mass × Food	79.187	70.643	0.781

**Figure 5:**
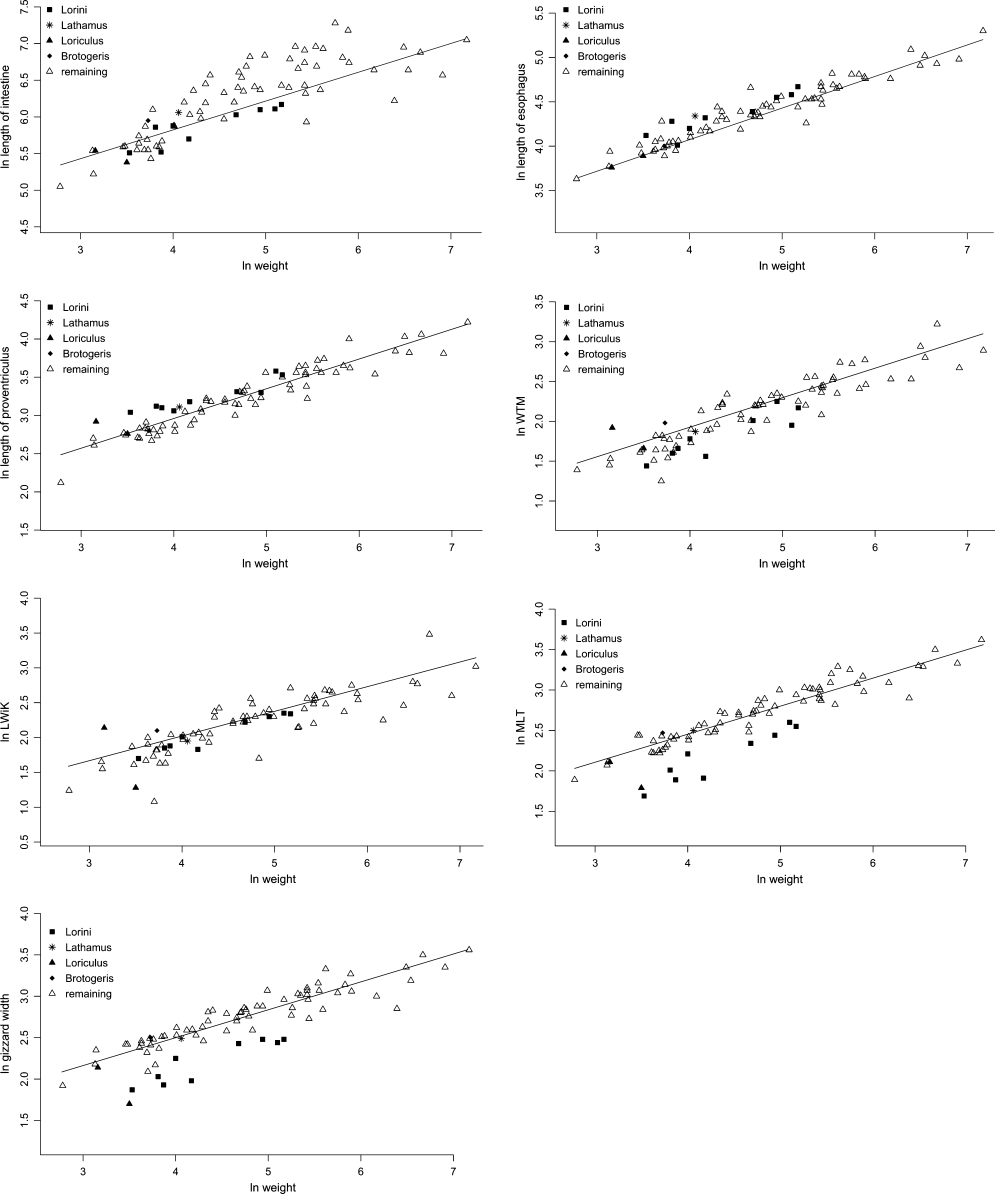
Natural-log transformed values of the different independent variables against natural-log transformed body masses including the regression lines of the best-fitting model. For all these traits, the data were best explained by an allometric relationship between the dependent and independent variables.

**Figure 6:**
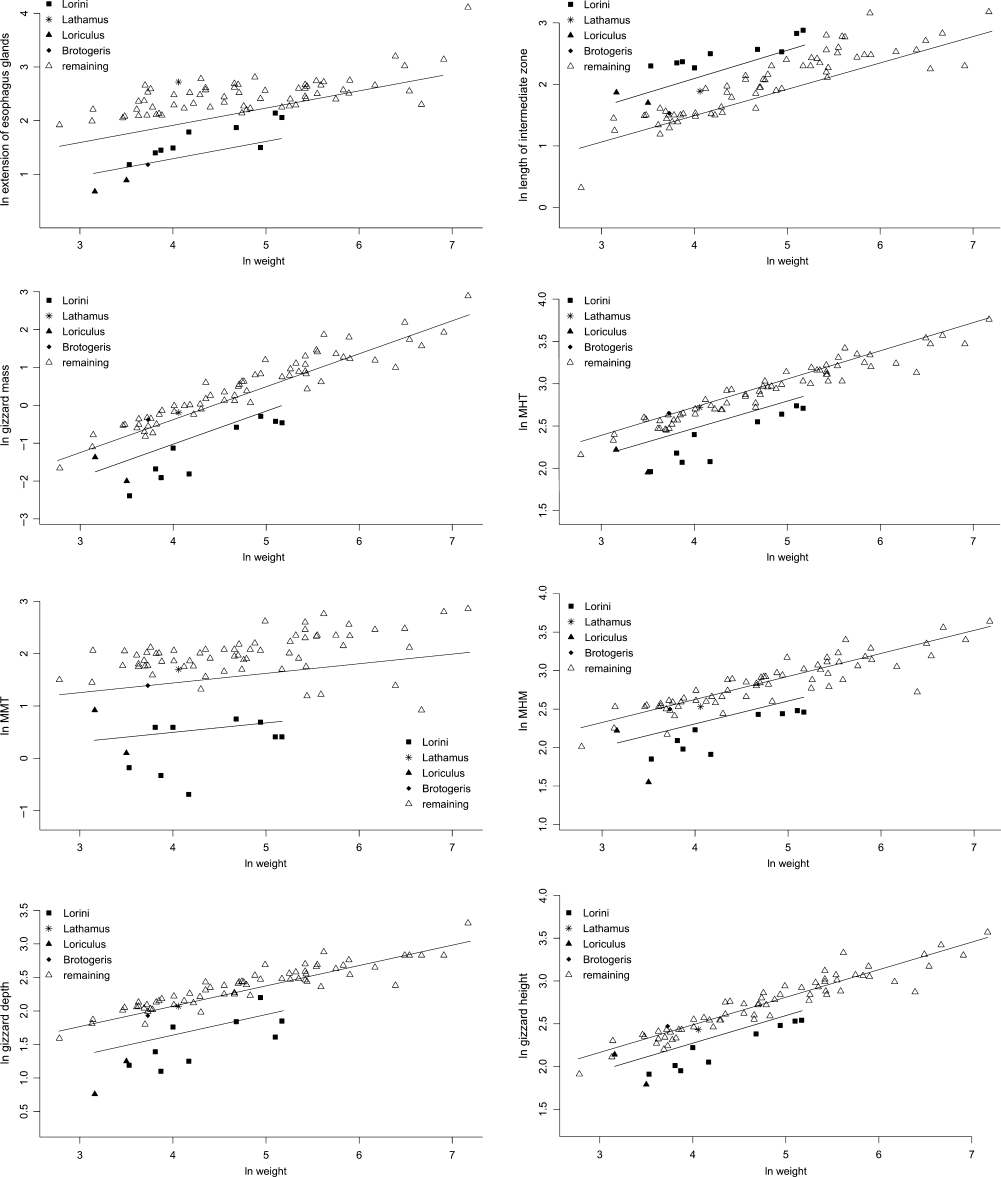
Natural-log transformed values of the different independent variables against natural-log transformed body masses including the regression lines of the best-fitting models. For all these traits, a model including the nectarivorous diet as a covariate with different intercepts but the same slope was considered as the best-fitting model.

For eight traits, a PGLS model including diet as a covariate with different intercepts but the same slope was considered to be the best-fitting model, while a simple allometric model best explained the data for the remaining traits (Tables 1, 2, Figs. 5, 6). The specialization to nectarivory led to a decrease in the extension of the esophagus glands, though the trait-value of *Lathamus* was seemingly more similar to the non-nectarivorous parrots and there was some variation within the lories. Furthermore, the length of the intermediate zone was found to be prolonged in nectarivorous parrots, though this was apparently not the case for *Brotogeris*.

**Table 2:** Regression parameters including *P*-values of the best-fitting model for the different traits of the digestive tract

Trait	Model		Value	SE	*t*-value	*P*-value
Length of intestine	Simple allometry	Intercept	4.251	0.251	16.957	0.000
		Slope	0.393	0.033	11.772	0.000
Length of esophagus	Simple allometry	Intercept	2.649	0.083	31.743	0.000
		Slope	0.356	0.014	25.711	0.000
Extension of esophagus glands	Mass + Food	Intercept	0.002	0.313	0.006	0.995
		Slope	0.321	0.044	7.227	0.000
		Intercept	0.627	0.141	4.431	0.000
Length of intermediate zone	Mass + Food	Intercept	0.258	0.256	1.008	0.317
		Slope	0.456	0.037	12.482	0.000
		Intercept	−0.221	0.115	−4.149	0.000
Length of proventriculus	Simple allometry	Intercept	1.404	0.120	11.717	0.000
		Slope	0.389	0.019	20.414	0.000
Gizzard height	Mass + Food	Intercept	0.984	0.116	8.481	0.000
		Slope	0.322	0.018	17.775	0.000
		Intercept	1.199	0.053	4.000	0.000
Gizzard width	Simple allometry	Intercept	1.155	0.158	7.302	0.000
		Slope	0.336	0.024	14.166	0.000
Gizzard depth	Mass + Food	Intercept	0.434	0.188	2.305	0.024
		Slope	0.303	0.027	11.301	0.000
		Intercept	0.864	0.085	5.071	0.000
Maximum gizzard height at main muscles (MHM)	Mass + Food	Intercept	1.115	0.159	7.027	0.000
		Slope	0.298	0.025	11.988	0.000
		Intercept	1.433	0.074	4.310	0.000
Gizzard thickness at main muscles (MMT)	Mass + Food	Intercept	−0.236	0.473	−0.499	0.619
		Slope	0.182	0.063	2.871	0.005
		Intercept	0.713	0.211	4.501	0.000
Gizzard lumen width including koilin layer (LWiK)	Simple allometry	Intercept	0.606	0.170	3.568	0.001
		Slope	0.354	0.030	11.831	0.000
Gizzard width at caudoventral thin muscle (WTM)	Simple allometry	Intercept	0.447	0.155	2.885	0.005
		Slope	0.370	0.024	15.300	0.000
Maximum gizzard height at thin muscle (MHT)	Mass + Food	Intercept	1.195	0.122	9.776	0.000
		Slope	0.320	0.018	17.920	0.000
		Intercept	1.391	0.055	3.542	0.000
Maximum gizzard lumen at thin muscle (MLT)	Simple allometry	Intercept	1.061	0.145	7.293	0.000
		Slope	0.348	0.022	16.081	0.000
Gizzard mass	Mass + Food	Intercept	−4.505	0.365	−12.352	0.000
		Slope	0.870	0.053	16.559	0.000
		Intercept	−3.854	0.165	3.947	0.000

The nectarivorous species clearly differed from the remaining parrots in the measurements of the gizzard, with the exception of the following measures: width of whole gizzard and its lumen, width at the caudoventral thin muscle (WTM) and the maximum lumen at thin muscles (MLT). *Brotogeris* and *Lathamus* were seemingly more like the nonnectarivorous parrots for all gizzard traits. This was apparently also the case for *Loriculus galgulus* in gizzard mass, gizzard height, gizzard thickness at main muscles (MMT), and maximum gizzard height at main muscle (MHM). The gizzard measurements of *Psittrichas fulgidus* showed a clear tendency to be more closely associated with the nectarivorous species than with the nonnectarivorous parrots.

In contrast, including diet as covariate in the model did not improve model-fit for the length of the esophagus or the intestine. While an intermediate value of *λ* was revealed for the esophagus length, the length of the intestine showed a strong phylogenetic signal with a value of *λ* close to one (Table 1). When diet was included in the model for the length of the proventriculus, the AIC was slightly lower for an ANCOVA with the same slopes but different intercepts compared to simple allometry (Fig. 5, Table 1). However, the more complex model was not substantially supported. Hence, the length of the proventriculus is best explained by simple allometry.

### Evolutionary trait shifts

For ten traits, we found a significant shift in trait evolution at the base of the lory radiation (Table 3). These included all traits for which a model with diet as covariate was the best-fitting model in the PGLS ANCOVA's. In addition to these traits, a significant shift in trait evolution at the base of the lories was found for gizzard width and MLT. When correcting for multiple comparisons, however, the shift in trait evolution was no longer significant for MLT and neither for gizzard height, gizzard width and MHM.

**Table 3:** *P*-values and drift speed of the test for rate shifts in morphological evolution at the base of the lory radiation

Trait	*P*-value	*σ*-value (drift speed)
Length of intestine	n.s.	0.9667
Length of esophagus	n.s.	0.5011
Extension of esophagus glands	2.26E-04	1.2912
Length of intermediate zone	1.30E-04	1.0891
Length of proventriculus	n.s.	0.6343
Gizzard height	0.0058	0.6319
Gizzard width	0.021	0.756
Gizzard depth	2.99E-04	0.8304
Maximum gizzard height at main muscles (MHM)	0.0254	0.9091
Gizzard thickness at main muscles (MMT)	2.00E-06	1.7508
Gizzard lumen width including koilin layer (LWiK)	n.s.	1.2635
Gizzard width at caudoventral thin muscle (WTM)	n.s.	0.8016
Maximum gizzard height at thin muscle (MHT)	0.0011	0.5496
Maximum gizzard lumen at thin muscle (MLT)	0.0091	0.6638
Gizzard mass	6.86E-05	1.5585

## Discussion

### Phenotype–environment correlations

The gastrointestinal tract may be considered one of the major interfaces between an organism and its environment, mediating their interactions (Karasov 1990). Several interspecific studies on other bird groups have shown that the size of the gut is related to diet and that the morphology of the gastrointestinal tract often reflects the birds' feeding strategies (Ricklefs 1996; Battley and Piersma 2005; Caviedes-Vidal et al. 2007; Lavin and Karasov 2008). Moreover, a direct influence of feeding strategies on the structures, functionality and physiology of the digestive tract has been shown in other vertebrates such as mammals (e.g., Schieck and Millar 1985; Korn 1992; Lovegrove 2010), amphibians and reptiles (e.g., Stevens and Hume 1995; O'Grady et al. 2005), and fish (e.g., German and Horn 2006; Wagner et al. 2009). However, it has to be considered that biological structures are not only fine tuned to their functional demands by natural selection but are also influenced by phylogenetic history and biochemical and mechanical constraints (Raia et al. 2010).

Within parrots, we have shown here that nectarivory is associated with reduced extension of the glands in the lower part of the esophagus below the crop (Pars thoracica). These glands produce a mucous secretion which helps hard ingesta to glide through the glandular stomach (proventriculus) and reduces the risk of mechanical damage to the latter (Güntert 1981). It can be expected that parrot species eating exclusively soft or liquid food evolve reductions of these glands within the esophagus, and our data corroborate this. On the other hand, we could not find any indication that the nectarivorous parrots have a longer esophagus as was proposed for the lories by Güntert (1981).

Between the glandular stomach (proventriculus) and the gizzard (muscular stomach, ventriculus), there is an intermediate zone characterized by the absence of compound glands of the former and absence of the koilin layer of the latter (Ziswiler and Farner 1972). This intermediate zone has the function of a storage space, where the proteolytic enzyme pepsin from the proventriculus can react with ingesta (Güntert 1981). We found the nectarivorous parrots to have a longer intermediate zone compared to the remaining parrots, even though the trait values of one nectarivorous taxon, *Brotogeris*, were more similar to the non-nectarivorous species. The intermediate zone appears to play an important role in the digestion of pollen (Güntert 1981) and a prolonged intermediate zone may be an adaptation to optimize the extraction of amino acids from pollen grains. Pollen grains have a high protein content, with their interior (protoplast) consisting of diverse amino acids (van Tets and Hulbert 1999; Gartrell and Jones 2001). Acidifications of pollen grains in the proventriculus may be important so that their contents can be extruded and digested, whereas mechanical break-up of pollen grains in the gizzard does not seem to be important (Gartrell and Jones 2001). The amount of energy extracted from meals can be enhanced by increasing the retention time (McWhorter et al. 2009). A prolongation of the intermediate zone can thus increase the rate of protein digestion as it increases the retention time of pollen grains, which seems to be the case in nectarivorous parrots. Pollen ingestion may require less energy than feeding on insects as an additional amino acid supply, because nectarivorous birds will encounter pollen while feeding on nectar (Nicolson and Fleming 2003).

Besides being a storage organ, the gizzard functions as an organ of mechanical digestion, the site of preliminary acid proteolytic digestion, and a filter for indigestible material (Ziswiler and Farner 1972). During contraction of the gizzard, the thick muscles close up, narrowing the lumen to a thin cleft and forcing the contents into two pouches (cranial and caudal sac, cf. Fig. 2) that lie under the thin muscles (McLelland 1979). Species feeding on a soft diet do not need the grinding function to break down their food, and can be expected to evolve reduced gizzard musculature (Steinbacher 1934; McLelland 1979). Indeed, the nectarivorous parrots differed from the remaining parrots by having less developed gizzard muscles. In contrast, a simple allometric relationship best explained the width of the whole gizzard and its lumen as well as its width at the caudoventral thin muscle and the maximum lumen at the thin muscles. The thin muscles act antagonistically to the main muscles and have no grinding function. Therefore, they are not expected to be developed more strongly in species relying on the grinding function of the gizzard. In congruence with our results, Richardson and Wooller (1990) also found two species of lories (*Glossopsitta porphyrocephala*, *Trichoglossus haematodus*) to have smaller and less muscular gizzards than four nonnectarivorous parrot species of Australia (*Melopsittacus undulatus*, *Barnardius zonarius*, *Neopsephotus bourkii*, *Platycercus icterotis*). Interestingly, we found that the reportedly mainly frugivorous Pesquet's parrot *Psittrichas fulgidus* (Collar 1997) shared a similarly reduced muscularity with the nectarivorous parrots. On the other hand, the blue-crowned hanging-parrot *Loriculus galgulus* did not show an overall reduced muscularity. This may correspond to the higher amount of seeds in its diet compared *to L. philippensis* (Homberger 1980). Similarly, gizzard measurements of *Lathamus discolor* and *Brotogeris jugularis* clustered with the non-nectarivorous parrots, and these two species were not found to have overall reduced gizzard muscularity either. Other studies also found *Lathamus discolor* to have retained the muscular gizzard of a granivorous species (Güntert and Ziswiler 1972; Gartrell et al. 2000). This may allow this species to feed on harder food when nectar and pollen are rare (Gartrell et al. 2000). Gizzard dimensions also vary in other passerines according to diet, with longer gizzards in seed- than in fruit- and insect-eaters and thicker muscular and glandular layers in insect- compared to fruit- and seed-eaters (Ricklefs 1996). Smaller gizzards with a reduced muscularity were also found in the nectarivorous honeyeaters (Meliphagidae) compared to similar-sized passerines (Richardson and Wooller 1986); however, phylogenetic nonindependence was not controlled for in that study.

Chemical digestion of food principally takes place in the intestine (Ziswiler and Farner 1972). Richardson and Wooller (1990) found two species of lories (*Glossopsitta porphyrocephala*, *Trichoglossus haematodus*) to have shorter intestines compared with four non-nectarivorous parrot species of Australia (*Melopsittacus undulatus*, *Barnardius zonarius*, *Neopsephotus bourkii*, *Platycercus icterotis*). This was explained as a consequence of sugars in nectar needing less processing in the intestine than other food. In contrast, we could not find any indication for shorter intestines of nectarivorous parrots. In a broad comparative study of birds, Lavin et al. (2008) did not find any significant effect of diet on small intestine length either (Lavin et al. 2008). However, this result is only partly comparable with ours, since we measured the whole lengths of the intestine owing to the difficulty of clearly distinguishing the small and the large intestine in parrots due to lack of caeca (Ziswiler and Farner 1972; Güntert 1981). In addition to lengths, intestine function depends *inter alia* on volume, surface area, villi, and microvilli area as well as enzymatic activity (Ricklefs 1996; Lavin et al. 2008; McWhorter et al. 2009). Moreover, the efficiency of digestion in the intestine may be influenced by the passive absorption of hydrosoluble compounds through the paracellular pathway. This is prominent in birds and may be especially important for nectarivores because they have to deal with large amounts of sugar in their diet (Karasov and Cork 1994; Napier et al. 2008; McWhorter et al. 2009). In general, birds have a lower nominal surface area of the intestine and a shorter small intestine as well as shorter digestive retention times than mammals; however, their higher passive absorption compared to mammals may compensate for this (McWhorter et al. 2009). This may render predictions about the intestine dimensions in relation to diet more difficult.

In conclusion, our analyses showed that nectarivorous parrots differ, after correction for phylogenetic nonindependence, from the remaining parrots in several traits of the digestive tract. Hence, we uncovered significant phenotype–environment correlations for the prolongation of the intermediate zone, the reduction of gizzard muscularity and the reduction of glands in the esophagus. The similarity in these trait features among some of the different nectarivorous groups is an indication of parallel evolution under the same or similar environmental conditions, that is, the shift to a nectarivorous diet, and implies that natural selection was the main driving force (cf. Losos et al. 1998; Schluter et al. 2004; Colosimo et al. 2005). Moreover, functional considerations suggest that the adaptations in the intermediate zone of nectarivorous parrots (probably except *Brotogeris*) allow them to rely effectively on nectar as a food source, and thus implying evidence for trait utility (Schluter 2000).

### Phenotypic flexibility of the gastrointestinal tract

The size, structure, and functional characteristics of the gastrointestinal tract of birds can reversibly change within the lifetime of a bird (phenotypic flexibility, sensu Piersma and Drent (2003)) as a fast adaptive response to current functionality demands caused by environmental changes or circannual endogenous control (Starck 1999a,b1999b; Piersma and Drent 2003; Starck and Rahmaan 2003; Battley and Piersma 2005; McWhorter et al. 2009). As pointed out by Lavin et al. (2008), comparative studies like ours have the limitation that species were not analyzed under common-garden conditions and thus it is not possible to assess to what extent the variation found among species is influenced by phenotypic flexibility and plasticity at the individual level. However, the individuals analyzed in this study all stem from captivity, where more stable conditions than in nature can be expected, thus mirroring a common-garden experiment. Furthermore, the inclusion of several individuals for most species and the wide range of body sizes among species considered certainly minimized the effect of intraspecific variation. There is additionally some evidence that phenotypic flexibility of the gastrointestinal tract is limited in parrots (cf. Güntert 1981). All individuals of *Lathamus* for example analyzed in this study were fed with a nectar-alternative, but they retained the partly muscular gizzard similar to that of a granivorous species, and the features of their gastrointestinal tract did not appear to differ from wild specimens analyzed by Gartrell et al. (2000). Nevertheless, further studies, preferably on wild birds, are needed to document the interplay between natural selection, plasticity and potential flexibility in features of the digestive tract in different parrot species.

### Evolutionary trait shifts and species proliferation

We found that in the lories, the diet shift to nectarivory was associated with a significant shift in morphological evolution, chiefly of several gizzard traits, at the base of their radiation, implying a trait shift or evolutionary jump.

The lories have diversified into an exceptionally species-rich clade (Schweizer et al. 2011) and their diet shift might thus be considered to be an evolutionary key innovation. Nectar may have provided a spatially widespread underutilized niche and this may have allowed the lories to expand their ranges and to colonize even remote oceanic islands, which may have fostered allopatric speciation. Even today, congeneric species of the lories generally do not overlap geographically (Collar 1997). Sympatry within genera is found in eastern Australia, New Guinea and Wallacea, regions with a complex and composite environmental and geological history with several potential vicariance opportunities in the past (Hall 2002; Esselstyn et al. 2009; Byrne et al. 2011; Deiner et al. 2011). However, this ecological expansion was not followed by further significant ecological specializations within the radiation of the lories. Similar to honeyeaters, the other highly nectarivorous and species-rich bird-group of Australasia (Newton 2003), lories are generalized flower visitors and their ecological relationships with plants are not as specialized as those of hummingbirds or sunbirds (Fleming and Muchhala 2008). Avian pollinator assemblages differ regionally and the evolutionary specializations between nectar-feeding birds and their food-plants are strongest in the Neotropics, decreasing through Africa and South Asia to Southeast Asia and Australasia (Fleming and Muchhala 2008). The co-evolution of specialized plant-pollinator relationships may take time. While the hummingbirds split from their closest relatives in the Eocene (about 50 Ma) or even earlier (Brown et al. 2008; Pratt et al. 2009) with a major radiation after 20 Ma (Jetz et al. 2012), the lories split from their common ancestor with *Melopsittacus* only in the middle Miocene (about 15 Ma, Schweizer et al. 2011). Hummingbirds certainly had more time to co-evolve with plants than did lories. However, the similarly specialized sunbirds are likely to be younger than hummingbirds, and the evolutionary diversification of the generalist honeyeaters started at a similar age (Eocene) (Barker et al. 2004). Thus, explanations other than time may account for the low ecological specialization of Australasian nectarivorous birds compared with sunbirds and hummingbirds. Specific interactions between plants and pollinators are only likely to evolve when floral resources are spatially and temporally predictable (e.g., Waser et al. 1996). While this seems to be the case in the Neotropics, flowering of eucalypts in Australia varies in space and time, and trees in lowland and montane Papua New Guinea commonly have non-annual flowering patterns (Fleming and Muchhala 2008). Birds feeding on them may hence not be able to afford to specialize. This may account for the low specialization in plant–pollinator relationships of Australasian nectarivorous birds and may explain the lack of evolution of plant-specific specializations among the lories after their shift to a nectarivorous diet.

In conclusion, the key innovation of the lories allowed an expansion into a new adaptive zone and we hypothesize that the subsequent species proliferation may have essentially been nonadaptive through allopatric speciation. The lories may thus be considered an example of a nonadaptive radiation (Rundell and Price 2009). It is possible that the ecological opportunity provided by their key innovation did not trigger an adaptive radiation because of the unpredictable nature of the new resource. The key innovation nevertheless promoted significant lineage diversification through allopatric partitioning of the same broad new niche. Although other parrot groups switched to a nectarivorous diet, this did not increase their diversification rates and species richness compared to other parrots (Schweizer et al. 2011). Various factors may have inhibited an increased rate of cladogenesis in *Lathamus, Brotogeris,* and *Loriculus* following their change to a nectarivorous diet. Such factors can include developmental or genetic constraints, but also ecological circumstances like interspecific competition or the lack of opportunities for allopatric speciation. Hence, an evolutionary innovation does not necessarily lead to increased diversification (Vermeij 2001; Price et al. 2010). The question as to which factors hampered increased species proliferation in other nectarivorous parrots will be an interesting avenue for future research.

## Supporting Information

**Appendix S1.** Mathematical Appendix.

**Table S1.** Number of individuals analyzed for each morphological trait.

**Table S2.** Species sampled, Museum and collection number, GenBank Accession numbers for the four genes analyzed.

## References

[b1] Akaike H (1974). New look at statistical-model identification. IEEE Trans. Automat. Contr.

[b2] Akester AR (1986). Structure of the glandular layer and koilin membrane in the gizzard of the adult domestic fowl (*Gallus gallus domesticus*. J. Anat.

[b3] Barker FK, Cibois A, Schikler P, Feinstein J, Cracraft J (2004). Phylogeny and diversification of the largest avian radiation. Proc. Natl Acad. Sci. USA.

[b4] Battley PF, Piersma T, Starck JM, Wang T (2005). Adaptive interplay between feeding ecology and features of the digestive tract in birds. Physiological and ecological adaptations to feeding in vertebrates.

[b5] Bawa KS (1990). Plant-pollinator interactions in tropical rain-forests. Ann. Rev. Ecol. Syst.

[b6] Blomberg SP, Garland T, Ives AR (2003). Testing for phylogenetic signal in comparative data: behavioral traits are more labile. Evolution.

[b7] Brice AT (1992). The essentiality of nectar and arthropods in the diet of the Anna hummingbird (*Calypte anna*. Comp. Biochem. Physiol. A.

[b8] Brown JH, Calder WA, Kodricbrown A (1978). Correlates and consequences of body size in nectar feeding birds. Am. Zool.

[b9] Brown JW, Rest JS, Garcia-Moreno J, Sorenson MD, Mindell DP (2008). Strong mitochondrial DNA support for a Cretaceous origin of modern avian lineages. BMC Biol.

[b10] Burnham KP, Anderson DR (2002). Model selection and mulitimodel inference.

[b11] Byrne M, Steane DA, Joseph L, Yeates DK, Jordan GJ, Crayn D (2011). Decline of a biome: evolution, contraction, fragmentation, extinction and invasion of the Australian mesic zone biota. J. Biogeogr.

[b12] Casotti G, Richardson KC (1993). A qualitative analysis of the kidney structure of meliphagid honeyeaters from wet and arid environments. J. Anat.

[b13] Casotti G, Beuchat CA, Braun EJ (1998). Morphology of the kidney in a nectarivorous bird, the Anna's hummingbird *Calypte anna*. J. Zool.

[b14] Caviedes-Vidal E, McWhorter TJ, Lavin SR, Chediack JG, Tracy CR, Karasov WH (2007). The digestive adaptation of flying vertebrates: high intestinal paracellular absorption compensates for smaller guts. Proc. Natl Acad. Sci. USA.

[b15] Churchill DM, Christensen P (1970). Observations on pollen harvesting by brush-tongued lorikeets. Aust. J. Zool.

[b16] Collar NJ, del Hoyo J, Elliott A, Sargatal J (1997). Family Psittacidae (parrots). Handbook of the birds of the world.

[b17] Colosimo PF, Hosemann KE, Balabhadra S, Villarreal G, Dickson M, Grimwood J (2005). Widespread parallel evolution in sticklebacks by repeated fixation of ectodysplasin alleles. Science.

[b18] Deiner K, Lemmon AR, Mack AL, Fleischer RC, Dumbacher JP (2011). A passerine bird's evolution corroborates the geologic history of the island of New Guinea. PLoS ONE.

[b19] Downs CT (2004). Some preliminary results of studies on the bill and tongue morphology of Gurney's sugarbird and some southern African sunbirds. Ostrich.

[b20] Esselstyn JA, Timm RM, Brown RM (2009). Do geological or climatic processes drive speciation in dynamic archipelagos? The tempo and mode of diversification in Southeast Asian shrews. Evolution.

[b21] Feinsinger P, Colwell RK (1978). Community organization among Neotropical nectar-feeding birds. Am. Zool.

[b22] Fleming TH, Muchhala N (2008). Nectar-feeding bird and bat niches in two worlds: pantropical comparisons of vertebrate pollination systems. J. Biogeogr.

[b23] Forshaw JM (1989). Parrots of the world.

[b24] Freckleton RP (2009). The seven deadly sins of comparative analysis. J. Evol. Biol.

[b25] Freckleton RP, Harvey PH, Pagel M (2002). Phylogenetic analysis and comparative data: a test and review of evidence. Am. Nat.

[b26] Garland T, Ives AR (2000). Using the past to predict the present: confidence intervals for regression equations in phylogenetic comparative methods. Am. Nat.

[b27] Gartrell BD (2000). The nutritional, morphologic, and physiologic bases of nectarivory in Australian birds. J. Avian Med. Surg.

[b28] Gartrell BD, Jones SM (2001). Eucalyptus pollen grain emptying by two Australian nectarivorous psittacines. J. Avian Biol.

[b29] Gartrell BD, Jones SM, Brereton RN, Astheimer LB (2000). Morphological adaptations to nectarivory of the alimentary tract of the swift parrot *Lathamus discolor*. Emu.

[b30] German DP, Horn MH (2006). Gut length and mass in herbivorous and carnivorous prickleback fishes (Teleostei: Stichaeidae): ontogenetic, dietary, and phylogenetic effects. Mar. Biol.

[b31] Grafen A (1989). The phylogenetic regression. Philos. Trans. R. Soc. B.

[b32] Grafen A (1992). The uniqueness of the phylogenetic regression. J. Theor. Biol.

[b33] Güntert M (1981). Morphologische Untersuchungen zur adaptiven Radiation des Verdauungstraktes bei Papageien (Psittaci). Zool. Jahrb. Abt. Anat. Ontog. Tiere.

[b34] Güntert M, Ziswiler V (1972). Konvergenzen in der Struktur von Zunge und Verdauungstrakt nektarfressender Papageien. Rev. Suisse Zool.

[b35] Hall TA (1999). BioEdit, a user-friendly biological sequence alignment editor and analysis 750 program for Windows 95/98/NT. Nucleic Acids Symp. Ser.

[b36] Hall R (2002). Cenozoic geological and plate tectonic evolution of SE Asia and the SW Pacific: computer-based reconstructions, model and animations. J. Asian Earth Sci.

[b37] Homberger DG (1980). Funktionell- morphologische Untersuchungen zur Radiation der Ernährungs- und Trinkmethoden der Papageien (Psittaci). Bonn. Zool. Monogr.

[b38] Housworth EA, Martins EP, Lynch M (2004). The phylogenetic mixed model. Am. Nat.

[b39] Huelsenbeck JP, Larget B, Swafford D (2000). A compound poisson process for relaxing the molecular clock. Genetics.

[b40] Hunter JP (1998). Key innovations and the ecology of macroevolution. Trends Ecol. Evol.

[b41] Jetz W, Thomas GH, Joy JB, Hartmann K, Mooers AO (2012). The global diversity of birds in space and time. Nature.

[b42] Joseph L, Toon A, Schirtzinger EE, Wright TF (2011). Molecular systematics of two enigmatic genera *Psittacella* and *Pezoporus* illuminate the ecological radiation of Australo-Papuan parrots (Aves: Psittaciformes). Mol. Phylogenet. Evol.

[b43] Karasov WH (1990). Digestion in birds: chemical and physiological determinants and ecological implications. Stud. Avian. Biol.

[b44] Karasov WH, Cork SJ (1994). Glucose-absorption by a nectarivorous bird - the passive pathway is paramount. Am. J. Physiol.

[b45] Kassen R, Schlichting CD, Mousseau TA (2009). Toward a general theory of adaptive radiation: insights from microbial experimental evolution. Year in evolutionary biology 2009.

[b46] Korn H (1992). Intestine lengths of Southern Africa savanna rodents and insectivores - intraspecific and interspecific comparisons. J. Zool.

[b47] Landis MJ, Schraiber JG, Liang M (2012). Phylogenetic analysos uisng Lévy processes: finding jumps in the evolution of continuous trats. Syst. Biol.

[b48] Lavin SR, Karasov WH (2008). Allometry of paracellular absorption in birds. Physiol. Biochem. Zool.

[b49] Lavin SR, Karasov WH, Ives AR, Middleton KM, Garland T (2008). Morphometrics of the avian small intestine compared with that of nonflying mammals: a phylogenetic approach. Physiol. Biochem. Zool.

[b50] Losos JB, Jackman TR, Larson A, de Queiroz K, Rodriguez-Schettino L (1998). Contingency and determinism in replicated adaptive radiations of island lizards. Science.

[b51] Lovegrove BG (2010). The allometry of rodent intestines. J. Comp. Physiol. B.

[b52] Lüttge U, Lüttge U, Pitman MG (1976). Chemical composition of nectars. Transport in plants II. Tissue and organs. Part B.

[b53] Martínez del Rio C, Chivers D, Langer P (1994). Nutritional ecology of fruit-eating and flower-visiting birds and bats. The digestive system in mammals: food form and function.

[b54] Martins EP, Hansen TF (1997). Phylogenies and the comparative method: a general approach to incorporating phylogenetic information into the analysis of interspecific data. Am. Nat.

[b55] Mayr E (1931). Birds collected during the Whitney South Sea expedition XIII. Am. Mus. Novit.

[b56] McLelland J, King AS, McLelland J (1979). Digestive system. Form and function in birds.

[b57] McWhorter TJ, Caviedes-Vidal E, Karasov WH (2009). The integration of digestion and osmoregulation in the avian gut. Biol. Rev.

[b58] Napier KR, McWhorter TJ, Fleming PA (2008). Mechanism and rate of glucose absorption differ between an Australian honeyeater (Meliphagidae) and a lorikeet (Loriidae). J. Exp. Biol.

[b59] Newton I (2003). The speciation and biogeography of birds.

[b60] Nicolson SW, Fleming PA (2003). Nectar as food for birds: the physiological consequences of drinking dilute sugar solutions. Plant Syst. Evol.

[b61] O'Grady SP, Morando M, Avila L, Dearing MD (2005). Correlating diet and digestive tract specialization: examples from the lizard family Liolaemidae. Zoology.

[b62] Pagel M (1997). Inferring evolutionary processes from phylogenies. Zool. Scr.

[b63] Pagel M (1999). Inferring the historical patterns of biological evolution. Nature.

[b64] Paradis E, Claude J, Strimmer K (2004). APE: analyses of phylogenetics and evolution in R language. Bioinformatics.

[b65] Piersma T, Drent J (2003). Phenotypic flexibility and the evolution of organismal design. Trends Ecol. Evol.

[b66] Pinheiro J, Bates D, DebRoy S, Sarkar D, Team, T. R. C (2009).

[b67] Pratt RC, Gibb GC, Morgan-Richards M, Phillips MJ, Hendy MD, Penny D (2009). Toward resolving deep Neoaves phylogeny: data, signal enhancement, and priors. Mol. Biol. Evol.

[b68] Price SA, Wainwright PC, Bellwood DR, Kazancioglu E, Collar DC, Near TJ (2010). Functional innovations and morphological diversification in parrotfish. Evolution.

[b800] R Development Core Team (2009). R: A language and environment for statistical computing.

[b69] Raia P, Carotenuto F, Meloro C, Piras P, Pushkina D (2010). The shape of contention: adaptation, history, and contingency in ungulate mandibles. Evolution.

[b70] Rand AL, Rabor DS (1960). Birds of the Philippine Islands: Siquijor, Mount Malindang, Bohol, and Samar. Fieldiana. Zool.

[b71] Richardson KC, Wooller RD (1986). The structures of the gastrointestinal tracts of honeyeaters and other small birds in relation to their diets. Aust. J. Zool.

[b72] Richardson KC, Wooller RD (1990). Adaptation of the alimentary tract of some Australian lorikeets to a diet of pollen and nectar. Aust. J. Zool.

[b73] Ricklefs RE (1996). Morphometry of the digestive tracts of some passerine birds. Condor.

[b74] Rowley I, Del Hoyo J, Elliott A, Sargatal J (1998). Family Cacatuidae (cockatoos). Handbook of the birds of the world.

[b75] Rundell RJ, Price TD (2009). Adaptive radiation, nonadaptive radiation, ecological speciation and nonecological speciation. Trends Ecol. Evol.

[b76] Schieck JO, Millar JS (1985). Alimentary-tract measurements as indicators of diets of small mammals. Mammalia.

[b77] Schlamowitz R, Hainsworth FR, Wolf LL (1976). Tongues of sunbirds. Condor.

[b78] Schluter D (2000). The ecology of adaptive radiation.

[b79] Schluter D, Clifford EA, Nemethy M, McKinnon JS (2004). Parallel evolution and inheritance of quantitative traits. Am. Nat.

[b80] Schmidt-Nielsen K (1984). Scaling: why is animal size so important?.

[b81] Schuchmann KL, del Hoyo J, Elliott A, Sargatal J (1999). Family Trochilidae (hummingbirds). Handbook of the birds of the world.

[b82] Schweizer M, Seehausen O, Güntert M, Hertwig ST (2010). The evolutionary diversification of parrots supports a taxon pulse model with multiple trans-oceanic dispersal events and local radiations. Mol. Phylogenet. Evol.

[b83] Schweizer M, Seehausen O, Hertwig ST (2011). Macroevolutionary patterns in the diversification of parrots: effects of climate change, geological events and key innovations. J. Biogeogr.

[b84] Schweizer M, Güntert M, Hertwig ST (2013). Out of the Bassian province: historical biogeography of the Australasian platycercine parrots (Aves, Psittaciformes). Zool. Scr.

[b85] Sekercioglu CH (2006). Increasing awareness of avian ecological function. Trends Ecol. Evol.

[b86] Stamatakis A (2006). RAxML-VI-HPC: maximum likelihood-based phylogenetic analyses with thousands of taxa and mixed models. Bioinformatics.

[b87] Stamatakis A, Hoover P, Rougemont J (2008). A rapid bootstrap algorithm for the RAxML web servers. Syst. Biol.

[b88] Starck JM (1999a). Phenotypic flexibility of the avian gizzard: rapid, reversible and repeated chances of organ size in response to changes in dietary fibre content. J. Exp. Biol.

[b89] Starck JM (1999b). Structural flexibility of the gastro-intestinal tract of vertebrates - implications for evolutionary morphology. Zool. Anz.

[b90] Starck JM, Rahmaan GHA (2003). Phenotypic flexibility of structure and function of the digestive system of Japanese quail. J. Exp. Biol.

[b91] Steinbacher G (1934). Zur Kenntnis des Magens blütenbesuchender Pagageien. Ornithol. Monatsber.

[b92] Stevens CE, Hume ID (1995). Comparative physiology of the vertebrate digestive system.

[b93] Tavares ES, Baker AJ, Pereira SL, Miyaki CY (2006). Phylogenetic relationships and historical biogeography of Neotropical parrots (Psittaciformes: Psittacidae: Arini) inferred from mitochondrial and nuclear DNA sequences. Syst. Biol.

[b94] Temeles EJ, Kress WJ (2003). Adaptation in a plant-hummingbird association. Science.

[b95] van Tets IG, Hulbert AJ (1999). A comparison of the nitrogen requirements of the eastern pygmy possum, *Cercartetus nanus*, on a pollen and on a mealworm diet. Physiol. Biochem. Zool.

[b96] van Tets IG, Nicolson SW (2000). Pollen and the nitrogen requirements of the lesser double-collared sunbird. Auk.

[b97] Uyeda JC, Hansen TF, Arnold SJ, Pienaar J (2011). The million-year wait for macroevolutionary bursts. Proc. Natl Acad. Sci. USA.

[b98] Vermeij GJ (1995). Economics, volcanos, and phanerozoic revolutions. Paleobiology.

[b99] Vermeij GJ (2001). Innovation and evolution at the edge: origins and fates of gastropods with a labral tooth. Biol. J. Linn. Soc.

[b100] Wagner CE, McIntyre PB, Buels KS, Gilbert DM, Michel E (2009). Diet predicts intestine length in Lake Tanganyika's cichlid fishes. Funct. Ecol.

[b101] Waser NM, Chittka L, Price MV, Williams NM, Ollerton J (1996). Generalization in pollination systems, and why it matters. Ecology.

[b102] Wright TF, Schirtzinger EE, Matsumoto T, Eberhard JR, Graves GR, Sanchez JJ (2008). A multilocus molecular phylogeny of the parrots (Psittaciformes): support for a Gondwanan origin during the Cretaceous. Mol. Biol. Evol.

[b103] Yoder JB, Clancey E, Des Roches S, Eastman JM, Gentry L, Godsoe W (2010). Ecological opportunity and the origin of adaptive radiations. J. Evol. Biol.

[b104] Ziswiler V (1967). Vergleichende morphologische Untersuchungen am Verdauungstrakt körnerfressender Singvögel zur Abklärung ihrer systematischen Stellung. Zool. Jb. Syst.

[b105] Ziswiler V, Farner DS, Farner DS, King JR (1972). Digestion and the digestive system. Avian biology.

