# Supplementary material for: Parallel adaptations to nectarivory in parrots, key innovations and the diversification of the Loriinae
Source: Ecol Evol. 2014 Jun 16;4(14):2867–83. doi: 10.1002/ece3.1131 (PMC4130445; doi:10.1002/ece3.1131)
Supplement: Supplementary file 1 — Appendix S1. Mathematical Appendix. [file ece30004-2867-SD1.pdf]

ONLINE SUPPORTING INFORMATION
Appendix S1

We consider the general linear model

$$\mathbf{y} = \mathbf{A}\beta_{12} + \mathbf{b}\beta_3 + \epsilon. \tag{1}$$

Here, $\mathbf{y} = (y_1, \ldots, y_N)$ are the logarithmic trait values, $\mathbf{A}$ is the $N \times 2$-matrix

$$\mathbf{A} = \begin{pmatrix} 1 & x_1 \\ 1 & x_2 \\ \vdots & \vdots \\ 1 & x_N \end{pmatrix},$$

where $x_1, \ldots, x_N$ are the logarithmic size values. The vector

$$\mathbf{b} = (1, 1, \ldots, 1, 0, \ldots, 0)'$$

contains ones at the K entries corresponding to the subgroup in question and zeros elsewhere. $\beta_{12} = (\beta_1, \beta_2)'$ and β_3 are the unknown model parameters, and the error term $\epsilon \sim \mathcal{N}(\mathbf{0}, \sigma^2\Sigma)$ is non-degenerate multivariate normal with covariance matrix $\sigma^2\Sigma$ where σ^2 is an unknown factor whereas the matrix Σ is known. Setting $\mathbf{X} = [\mathbf{A}|\mathbf{b}]$ and $\beta = (\beta_1, \beta_2, \beta_3)'$, we can rewrite the model (1) more conveniently as

$$\mathbf{y} = \mathbf{X}\beta + \epsilon. \tag{2}$$

We have

Theorem 1. *Let $\hat{\beta}_3$ the GLM estimator of β_3 and s^2 the GLM estimator of σ^2. Moreover, set*

$$\mathbf{R} = (0, 0, 1).$$

Under the null hypothesis $H_0 : \beta_3 = 0$ (no selection on the subgroup) the test statistic

$$\hat{T} := \frac{\hat{\beta}_3}{s\sqrt{\mathbf{R}(\mathbf{X}'\Sigma^{-1}\mathbf{X})^{-1}\mathbf{R}'}} \tag{3}$$

has the t_{N-3}-distribution.

Proof. Since Σ is positive definite and symmetric it has a symmetric and positive definite square root $\mathbf{Q}$, i.e. $\mathbf{Q}^2 = \Sigma$. Multiplying both sides of (2) with $\mathbf{Q}^{-1}$ we get the homoskedastic model

$$\mathbf{y}_0 = \mathbf{X}_0\beta + \epsilon_0, \tag{4}$$

where $\mathbf{y}_0 = \mathbf{Q}^{-1}\mathbf{y}$, $\mathbf{X}_0 = \mathbf{Q}^{-1}\mathbf{X}$, and $\epsilon_0 \sim \mathcal{N}(\mathbf{0}, \sigma^2\mathbf{I})$. For this we have the usual estimators

$$\hat{\beta} = (\mathbf{X}_0'\mathbf{X}_0)^{-1}\mathbf{X}_0'\mathbf{y}_0 = (\mathbf{X}'\Sigma^{-1}\mathbf{X})^{-1}\mathbf{X}'\Sigma^{-1}\mathbf{y}$$

and

$$\begin{aligned} s^2 &= \frac{1}{N-3}\mathbf{y}_0'\left(\mathbf{I} - \mathbf{X}_0(\mathbf{X}_0'\mathbf{X}_0)^{-1}\mathbf{X}_0'\right)\mathbf{y}_0 \\ &= \frac{1}{N-3}\left(\mathbf{y}'\Sigma^{-1}\mathbf{y} - \mathbf{y}'\Sigma^{-1}\mathbf{X}(\mathbf{X}'\Sigma^{-1}\mathbf{X})\mathbf{X}'\Sigma^{-1}\mathbf{y}\right). \end{aligned}$$

It follows from the theory in Ruud (2000), Chapter 11, that the test statistic

$$\hat{F} := \frac{1}{s^2}\left(\mathbf{R}(\mathbf{X}_0'\mathbf{X}_0)^{-1}\mathbf{R}'\right)^{-1}(\mathbf{R}\hat{\beta})^2$$

for the model (4) has the $F_{1,N-3}$-distribution under the null-hypothesis

$$H_0: \ \mathbf{R}\beta = \beta_3 = 0.$$

It is easy to check that

$$\hat{F} = \frac{\left(\mathbf{R}(\mathbf{X}'\Sigma^{-1}\mathbf{X})^{-1}\mathbf{R}'\right)^{-1}}{s^2}\hat{\beta}_3^2.$$

Since $\hat{T} = \hat{F}^{1/2}$ and $F_{1,N-3} \sim t_{N-3}^2$, the claim follows. □

A similar test statistic can be derived if not only a shift of intercept, but also a change of slope of the regression line for the subgroup is considered in the alternative hypothesis. In this case, we consider the general linear model

$$\mathbf{y} = \mathbf{A}\beta_{12} + \mathbf{b}\beta_3 + \mathbf{c}\beta_4 + \epsilon. \tag{5}$$

Notation is as above, the only new ingredient is

$$\mathbf{c} = (x_1, x_2, \ldots, x_K, 0, \ldots, 0)'$$

where the non-zero entries correspond to the subgroup in question. The parameter β_4 models a possible change in slope for the subgroup. Setting $\mathbf{X} = [\mathbf{A}|\mathbf{b}|\mathbf{c}]$ and $\beta = (\beta_1, \ldots, \beta_4)'$, we can again rewrite the model (6) more conveniently as

$$\mathbf{y} = \mathbf{X}\beta + \epsilon. \tag{6}$$

An argument analogous to the one given above yields

Theorem 2. *Let $\hat{\beta}$ the GLM estimator of β in (6) and s^2 the GLM estimator of σ^2. Moreover, set*

$$\mathbf{R} = \begin{pmatrix} 0 & 0 & 1 & 0 \\ 0 & 0 & 0 & 1 \end{pmatrix}.$$

Under the null hypothesis $H_0: \ \beta_3 = \beta_4 = 0$ (or equivalently $\mathbf{R}\beta = \mathbf{0}$) the test statistic

$$\hat{F} := \frac{1}{2s^2}\hat{\beta}'\mathbf{R}'\left(\mathbf{R}(\mathbf{X}'\Sigma^{-1}\mathbf{X})^{-1}\mathbf{R}'\right)^{-1}\mathbf{R}\hat{\beta} \tag{7}$$

has the $F_{2,N-4}$-distribution.

Literature

Ruud P. A. 2000. An Introduction to Classical Econometric Theory. Oxford University Press, Oxford, New York.
