# Supplementary material for: Parallel adaptations to nectarivory in parrots, key innovations and the diversification of the Loriinae
Source: Ecol Evol. 2014 Jun 16;4(14):2867–83. doi: 10.1002/ece3.1131 (PMC4130445; doi:10.1002/ece3.1131)
Supplement: Supplementary file 2 — Table S1. Number of individuals analyzed for each morphological trait. [file ece30004-2867-SD2.pdf]

ONLINE SUPPORTING INFORMATION

Table S1. Number of individuals analyzed for each morphological trait.

Species	length of intestine	length of esophagus	extension of esophagus glands	length of intermediate zone	length of pro-ventriculus	gizzard height	gizzard width	gizzard depth	maximum gizzard height at main muscles (MHM)	gizzard thickness at main muscles (MMT)	gizzard lumen width including koilin layer (LWiK)	gizzard width at caudoventral thin muscle (WTM)	maximum gizzard height at thin muscle (MHT)	maximum gizzard lumen at thin muscle (MLT)	gizzard mass
Agapornis canus	4	3	3	3	3	4	4	4	4	4	4	4	4	4	4
Agapornis fischeri	11	10	8	8	8	9	9	9	9	9	9	9	9	9	9
Agapornis lilianae	6	7	7	7	7	7	7	7	7	6	6	6	7	7	7
Agapornis nigrigenis	2	2	1	2	2	2	2	2	2	2	2	2	2	2	1
Agapornis roseicollis	5	5	3	4	4	4	4	4	4	4	4	4	4	4	4
Agapornis personatus	4	4	3	4	4	4	4	4	4	4	4	2	4	4	4
Alisterus chloropterus	1	1	1	1	1	2	2	2	2	2	2	2	2	2	2
Alisterus scapularis	6	6	6	5	5	6	6	6	6	6	6	6	6	6	5
Amazona aestiva	5	5	5	5	5	5	5	5	5	5	5	5	5	5	5
Amazona dufresniana	1	1	1	1	1	1	1	1	1	1	1	1	1	1	1
Amazona pretrei	1	1	1	1	1	1	1	1	1	1	1	1	1	1	1
Amazona xanthops	1	1	1	1	1	1	1	1	1	1	1	1	1	1	1
Anodohynchus hyacinthinus	1	1	1	1	1	1	1	1	1	1	1	1	1	1	1
Aprosmictus jonquillaceus	4	5	4	3	3	4	4	4	4	4	4	4	4	4	4
Ara ararauna	1	1	1	1	1	1	1	1	1	1	1	1	1	1	1
Aratinga leucophthalmus	1	1	1	1	1	1	1	1	1	1	1	1	1	1	1
Ara macao	1	1	1	1	1	1	1	1	1	1	1	1	1	1	1
Aratinga solstitialis	2	2	2	2	2	2	2	2	2	2	2	2	2	2	2
Barnardius zonarius	6	6	6	6	6	5	5	5	5	5	5	5	5	5	5
Barnardius barnardi	5	5	5	5	5	5	5	5	5	5	5	5	5	5	5
Brotogeris jugularis	5	5	5	5	5	5	5	5	5	5	5	4	5	5	4
Cacatua goffini	1	1	1	1	1	1	1	1	1	1	1	1	1	1	1
Cacatua moluccensis	1	1	1	1	1	1	1	1	1	1	1	1	1	1	1
Cacatua sulphurea	3	3	3	3	3	3	3	3	3	3	3	3	3	3	3
Chalcopsitta cardinalis	1	1	1	1	1	1	1	1	1	1	1	1	1	1	1
Charmosyna pulchella	7	7	6	7	7	6	6	6	6	6	6	6	6	6	6
Coracopsis vasa	2	2	2	2	2	2	2	2	2	2	2	2	2	2	2
Cyanoramphus auriceps	5	4	6	6	6	6	6	6	6	6	6	6	6	6	5
Cyanoramphus novaezelandiae	3	2	2	2	2	3	3	3	3	3	3	3	3	3	3
Cyclopsitta diophthalma	4	5	4	4	4	4	4	4	4	4	4	4	4	4	4
Deroptyus accipitrinus	1	1	1	1	1	1	1	1	1	1	1	1	1	1	1
Eclectus roratus	6	6	6	5	5	6	6	6	6	6	6	6	6	6	6
Eos cyanogenia	1	1	1	1	1	1	1	1	1	1	1	1	1	1	1
Eunymphicus (cornutus) cornutus	1	1	1	1	1	1	1	1	1	1	1	1	1	1	1
Eunymphicus (cornutus) uvaeensis	5	6	6	6	6	6	6	6	6	6	6	6	6	6	6
Forpus passerinus	11	11	10	10	11	11	11	11	11	11	11	11	11	11	10
Guaruba guarouba	2	2	2	2	2	2	2	2	2	2	2	2	2	2	2
Lathamus discolor	5	5	3	5	5	3	3	3	3	3	3	3	3	3	2
Loriculus galgulus	3	3	3	2	2	3	3	3	2	2	3	3	3	2	2
Loriculus philippensis	4	4	4	3	4	1	1	1	1	1	1	2	1	1	2
Lorius garrulus	5	5	5	4	4	4	4	4	4	4	4	4	4	4	4
Melopsittacus undulatus	6	6	6	4	4	7	7	7	7	7	7	6	7	7	6
Micropsitta finschii	4	4	4	4	4	4	4	4	4	4	4	4	4	4	4
Neophema chrysostoma	4	3	3	2	2	3	3	3	3	3	3	3	3	3	3
Neophema pulchella	5	7	7	7	7	6	6	6	6	6	6	6	6	6	6
Neophema splendida	10	10	12	12	12	11	11	11	11	11	11	10	11	11	11
Neopsephotos bourkii	7	7	8	7	7	8	8	8	8	8	8	7	8	8	8
Nestor notabilis	1	1	1	1	1	1	1	1	1	1	1	1	1	1	1
Northiella haematogaster	10	13	13	13	13	13	13	13	13	12	12	13	13	13	13
Phigys solitarius	7	8	5	7	7	2	2	2	2	2	2	2	2	2	3
Pionus maximiliani	1	1	1	1	1	1	1	1	1	1	1	1	1	1	1
Pionus menstruus	2	2	2	2	2	2	2	2	2	2	2	2	2	2	2
Platycercus caledonicus	5	5	5	5	5	5	5	5	5	5	5	5	5	5	5
Platycercus eximius	3	3	3	3	3	3	3	3	3	3	3	3	3	3	3
Platycercus flaveolus	3	2	2	2	2	3	3	3	3	3	3	2	2	2	2

ONLINE SUPPORTING INFORMATION

Table S1. Continued.

Species	length of intestine	length of esophagus	extension of esophagus glands	length of intermediate zone	length of pro-ventriculus	gizzard height	gizzard width	gizzard depth	maximum gizzard height at main muscles (MHM)	gizzard thickness at main muscles (MMT)	gizzard lumen width including koilin layer (LWiK)	gizzard width at caudoventral thin muscle (WTM)	maximum gizzard height at thin muscle (MHT)	maximum gizzard lumen at thin muscle (MLT)	gizzard mass
Platycercus venustus	5	5	5	5	5	5	5	5	5	5	5	5	5	5	5
Poicephalus gulielmi	1	1	1	1	1	1	1	1	1	1	1	1	1	1	1
Poicephalus senegalus	9	9	9	9	9	8	8	8	8	8	8	8	8	7	8
Polytelis alexandrae	6	7	6	6	6	7	7	7	7	7	7	6	7	7	6
Polytelis anthopeplus	1	1	1	1	1	1	1	1	1	1	1	1	1	1	1
Prioniturus luconensis	2	1	2	1	1	2	2	2	2	2	2	2	2	2	2
Prosopeia tabuensis	10	9	11	10	10	12	12	12	12	12	12	10	12	12	11
Psephotus chrysopterygius	5	4	4	4	4	4	4	4	4	4	4	4	4	4	4
Psephotus dissimilis	9	9	10	9	9	9	9	9	9	9	9	9	9	9	8
Psephotus haematonotus	4	3	3	4	4	4	4	4	4	4	4	3	4	4	4
Psittacula eupatria	2	2	2	2	2	2	2	2	2	2	2	2	2	2	2
Psephotus varius	4	5	5	5	5	5	5	5	5	5	5	4	5	5	4
Psittaculirostris desmarestii	2	3	2	2	2	2	2	2	2	2	2	2	2	2	2
Psittacus erithacus	6	5	6	6	6	6	6	6	6	6	6	6	6	6	6
Psitteuteles goldiei	5	5	5	6	6	4	4	4	4	5	4	4	4	4	5
Psittinus cyanurus	4	4	4	4	4	4	4	4	4	4	4	4	4	4	4
Psittrichas fulgidus	4	3	4	4	4	3	3	3	3	3	3	3	3	3	3
Purpureicephalus spurius	4	4	3	4	4	4	4	4	4	4	4	4	4	4	4
Tanygnathus megalorhynchus	2	2	2	2	2	2	2	2	2	2	2	2	2	2	2
Trichoglossus haematodus	16	16	16	16	16	12	12	12	12	12	12	12	12	12	11
Trichoglossus johnstoniae	7	7	7	7	7	7	7	7	7	7	7	7	7	7	7
Triclaria malachitacea	1	1	1	1	1	1	1	1	1	1	1	1	1	1	1
Vini australis	10	8	7	9	9	9	9	9	9	9	9	9	9	9	6
