# Supplementary material for: Parallel adaptations to nectarivory in parrots, key innovations and the diversification of the Loriinae
Source: Ecol Evol. 2014 Jun 16;4(14):2867–83. doi: 10.1002/ece3.1131 (PMC4130445; doi:10.1002/ece3.1131)
Supplement: Supplementary file 3 — Table S2. Species sampled, Museum and collection number, GenBank Accession numbers for the four genes analyzed. [file ece30004-2867-SD3.doc]

**ONLINE SUPPORTING INFORMATION**

**Table S2.** Species sampled, Museum and collection number, GenBank Accession numbers for the four genes analyzed.

|  | | | | | | |
| --- | --- | --- | --- | --- | --- | --- |
| Species | Museum/Collection | Collection nb. | c-mos | Rag-1 | Zenk | ND2 |
|  | | | | | | |
|  | | | | | | |
| *Agapornis canus* | NMBE | 1056201 | GQ505083 | GQ505191 | GQ505138 | - |
| *Agapornis fischeri* | NMBE | 1056202 | GQ505084 | GQ505192 | GQ505139 | - |
| *Agapornis lilianae* | NMBE | 1056205 | GQ505087 | - | - | - |
| *Agapornis nigrigenis* | NMBE | 1056203 | GQ505085 | GQ505193 | GQ505140 | - |
| *Agapornis roseicollis* | NMBE | 1056204 | GQ505086 | GQ505194 | GQ505141 | EU327596 |
| *Agapornis personatus* | NMBE | 1059098 | KJ817818 | KJ817821 | - | - |
| *Alisterus chloropterus* | NMBE | 1056207 | GQ505091 | GQ505199 | GQ505145 | - |
| *Alisterus scapularis* | NMBE | 1056206 | GQ505090 | GQ505198 | GQ505144 | - |
| *Amazona aestiva* | NMBE | 1056947 | JF807952 | JF807980 | JF807966 | AY194434 |
| *Amazona dufresniana* | NMBE | 1056948 | JF807953 | JF807981 | JF807967 | - |
| *Amazona pretrei* | NMBE | 1056949 | JF807954 | JF807982 | JF807968 | - |
| *Amazona xanthops* | NMBE | 1059099 | KJ817819 | KJ817822 | - | DQ143316 |
| *Anodohynchus hyacinthinus* |  |  | - | DQ143329 | - | DQ143311 |
| *Aprosmictus jonquillaceus* | NMBE | 1056208 | GQ505092 | GQ505200 | GQ505146 | - |
| *Ara ararauna* |  |  | - | DQ143340 | - | DQ143315 |
| *Aratinga leucophthalmus* |  |  | - | DQ143331 | - | DQ143298 |
| *Ara macao* | NMBE | 1056952 | JF807951 | JF807979 | JF807965 | EU327601 |
| *Aratinga solstitialis* |  |  | - | DQ143330 | - | DQ143317 |
| *Barnardius zonarius* | NMBE | 1056210 | GQ505095 | GQ505203 | GQ505149 | JQ066250 |
| *Barnardius barnardi* | NMBE | 1059100 | - | JX442402 | - | JX442398 |
| *Brotogeris jugularis* |  |  | - | - | - | EU327604 |
| *Cacatua goffini* |  |  | - | DQ143355 | - | DQ143323 |
| *Cacatua moluccensis* | NMBE | 1056236 | GQ505121 | GQ505232 | GQ505174 | - |
| *Cacatua sulphurea* |  |  | - | - | EU738913 | EU327605 |
| *Chalcopsitta cardinalis* |  |  | - | - | EU738919 | - |
| *Charmosyna pulchella* | NMBE | 1056241 | GQ505126 | GQ505237 | GQ505179 | JX442396 |
| *Coracopsis vasa* | UWBM | 85986/2004-001 | GQ505113 | GQ505223 | GQ505167 | EU327612 |
| *Cyanoramphus auriceps* | NMBE | 1056221 | GQ505104 | GQ505213 | GQ505158 | JX442387 |
| *Cyanoramphus novaezelandiae* | NMBE | 1056220 | GQ505103 | GQ505212 | GQ505157 | JQ066252 |
| *Cyclopsitta diophthalma* |  |  | GQ505130 | - | - | EU327616 |
| *Deroptyus accipitrinus* | NMBE | 1056951 | F807956 | JF807984 | JF807970 | EU327617 |
| *Eclectus roratus* | NMBE | 1056248 | GQ505135 | GQ505244 | GQ505187 | EU327619 |
| *Eos cyanogenia* | NMBE | 1056237 | GQ505122 | GQ505233 | GQ505175 | JQ066254 |
| *Eunymphicus (cornutus) cornutus* | NMBE | 1056223 | GQ505106 | GQ505215 | GQ505159 | JX442388 |
| *Eunymphicus( cornutus) uvaeensis* | NMBE | 1056224 | GQ505107 | GQ505216 | GQ505160 | JX442389 |
| *Forpus passerinus* |  |  | - | - | - | EU327625 |
| *Guaruba guarouba* | NMBE | 1056950 | JF807957 | JF807985 | JF807971 | EU327628 |
| *Lathamus discolor* | NMBE | 1056219 | GQ505102 | GQ505211 | GQ505156 | JX442385 |
| *Loriculus galgulus* | UWBM | 73841/2002-006 | GQ505089 | GQ505196 | - | EU327631 |
| *Loriculus philippensis* | ZMUC | 130608 | - | GQ505197 | GQ505143 | - |
| *Lorius garrulus* | NMBE | 1056240 | GQ505125 | GQ505236 | GQ505178 | JQ066256 |
| *Melopsittacus undulatus* | UWBM | 60748/1998-068 | - | GQ505222 | GQ505166 | EU327633 |
| *Micropsitta finschii* | UWBM | 66040/2000-022 | GQ505128 | GQ505240 | GQ505182 | EU327634 |
| *Neophema chrysostoma* | NMBE | 1056228 | GQ505111 | GQ505220 | GQ505164 | JX442392 |
| *Neophema pulchella* | NMBE | 1056226 | GQ505109 | GQ505218 | GQ505162 | JX442391 |
| *Neophema splendida* | NMBE | 1056225 | GQ505108 | GQ505217 | GQ505161 | JQ066253 |
| *Neopsephotos bourkii* | UWBM | 57542/1996-109 | GQ505112 | GQ505221 | GQ505165 | EU327639 |
| *Nestor notabilis* | NMBE | 1056242 | JF807958 | GQ505238 | GQ505180 | EU327641 |
| *Northiella haematogaster* | NMBE | 1056954 | JF807959 | JF807986 | JF807972 | JX442397 |
| *Phigys solitarius* |  |  | - | - | - | EU327646 |
| *Pionus maximiliani* |  |  | - | DQ143347 | - | EF517657 |
| *Pionus menstruus* | NMBE | 1056955 | JF807960 | JF807987 | JF807973 | EU327650 |
| *Platycercus caledonicus* | NMBE | 1056212 | GQ505097 | GQ505205 | GQ505151 | EU407679 |
| *Platycercus eximius* | NMBE | 1056213 | - | GQ505206 | GQ505152 | EU407711 |
| *Platycercus flaveolus* | NMBE | 1056215 | GQ505099 | GQ505208 | - | EU407696 |
| *Platycercus venustus* | NMBE | 1056214 | GQ505098 | GQ505207 | GQ505153 | JX442382 |
| *Poicephalus gulielmi* | FMNH | 390740 | JF807961 | JF807988 | JF807974 | - |
| *Poicephalus senegalus* | NMBE | 1056231 | - | GQ505227 | GQ505170 | - |
| *Polytelis alexandrae* | NMBE | 1056209 | GQ505093 | GQ505201 | GQ505147 | EU327653 |
| *Polytelis anthopeplus* | NMBE | 1056657 | GQ505094 | GQ505202 | GQ505148 | EU407716 |
| *Prioniturus luconensis* | NMBE | 1056247 | GQ505134 | - | - | EU327654 |
| *Prosopeia tabuensis* | NMBE | 1056252 | GQ505105 | GQ505214 | - | EU327656 |
| *Psephotus chrysopterygius* | NMBE | 1056953 | JF807963 | JF807991 | F807977 | JX442400 |
| *Psephotus dissimilis* | NMBE | 1056218 | GQ505101 | GQ505210 | GQ505155 | JX442384 |
| *Psephotus haematonotus* | NMBE | 1059101 | JX442404 | JX442403 | JX442379 | JX442399 |
| *Psittacula eupatria* | NMBE | 1056250 | GQ505137 | GQ505246 | GQ505189 | - |
| *Psephotus varius* | NMBE | 1056217 | GQ505100 | GQ505209 | GQ505154 | JX442383 |
| *Psittaculirostris desmarestii* | NMBE | 1056244 | GQ505131 | GQ505242 | GQ505184 | - |
| *Psittacus erithacus* | NMBE | 1056229 | GQ505115 | GQ505225 | GQ505168 | EU327661 |
| *Psitteuteles goldiei* | NMBE | 1056239 | GQ505124 | GQ505235 | GQ505177 | JQ066255 |
| *Psittinus cyanurus* | NMBE | 1056251 | - | GQ505247 | GQ505190 | - |
| *Psittrichas fulgidus* | NMBE | 1056243 | GQ505127 | GQ505239 | GQ505181 | EU327662 |
| *Purpureicephalus spurius* | NMBE | 1056211 | GQ505096 | GQ505204 | GQ505150 | JX442381 |
| *Tanygnathus megalorhynchus* | NMBE | 1056249 | GQ505136 | GQ505245 | GQ505188 | - |
| *Trichoglossus haematodus* | NMBE | 1059102 | - | KJ817823 | - | EU327671 |
| *Trichoglossus johnstoniae* | NMBE | 1056238 | GQ505123 | GQ505234 | GQ505176 | KJ817820 |
| *Triclaria malachitacea* | NMBE | 1056232 | GQ505117 | GQ505228 | GQ505171 | AY669486 |
| *Vini australis* |  |  | - | - | - | EU327672 |
| *Falco* |  |  | AY447974 | AY461399 | AF490155 | EU196361 |
| *Pitta* |  |  | AY056952 | AY057021 | EF568299 | GQ369692 |
|  | | | | | | |
